# Economic insecurity and depression among youth of color during the COVID-19 pandemic

**DOI:** 10.1186/s12889-025-24811-9

**Published:** 2025-11-25

**Authors:** Tasfia Jahangir, Marcia J. Ash, Melvin D. Livingston, Regine Haardörfer, Jannette Berkley-Patton, Briana Woods-Jaeger

**Affiliations:** 1 Emory University Rollins School of Public Health, Behavioral, Social and Health Education Sciences, 1518 Clifton Rd NE, Atlanta, GA 30322 USA; 2 University of Missouri-Kansas City, Kansas, MO USA

**Keywords:** Mental health, COVID-19, Economic insecurity, Racism, Youth

## Abstract

**Background:**

Communities of color have disproportionately faced burdens and losses during COVID-19, including greater economic instability and higher death rates. These overlapping stressors heightened the risk of negative mental health among youth of color. We relied on the Unified Macrotheory of Depression among Urban Black youth to examine associations among three indicators of economic insecurity – COVID-related financial insecurity, neighborhood income, and free/reduced-price lunch eligibility— and depressive symptoms, as well as the potential mediating roles of stress and loneliness.

**Methods:**

We analyzed cross-sectional survey data among 105 adolescents and young adults of color (majority Black) in Kansas City, Missouri. Participants reported on their experiences of financial insecurity, stress, loneliness and depressive symptoms measured variable path analysis.

**Results:**

COVID-related financial insecurity was significantly associated with stress, loneliness, and depressive symptoms. Significant indirect associations were observed from COVID-related financial insecurity to depression via both loneliness (standardized pathway coefficient = 0.088, SE = 0.045, 95% CI [0.018, 0.191]) and stress (standardized pathway coefficient = 0.074, SE = 0.04, 95% CI [0.017, 0.180]).

**Conclusion:**

Although the pandemic intensified acute stressors, these findings highlight deeper, long-standing structural inequities that contribute to the mental health challenges of youth of color and remain relevant in the post-pandemic period. The personal salience of COVID-related financial strain may have had a particularly strong psychological link during this period. Accordingly, we discuss implications for clinical, community, and policy interventions that address both acute financial disruptions and persistent structural conditions, such as financial insecurity, stress, and social isolation, in our population of interest.

## Introduction

There are persistent associations between financial insecurity and adverse mental health [1–6]. Prior research suggests that children may be especially vulnerable to the effects of financial insecurity, as healthy development is predicated on conducive social environments [7–20]. Financial insecurity is associated with both chronic and acute stress [20–26], which may be compounded by racism-related stressors among youth of color [27–32]. Together, racism and poverty-related stressors can culminate in both internalizing and externalizing mental health symptoms among youth of color [32].

Since the COVID-19 pandemic, depressive symptoms among adolescents doubled globally [33]. In the United States, the Surgeon General issued an advisory on “the devasting impact of the epidemic of loneliness and isolation” among youth [34]. Indeed, questionnaires completed by youth and parents in 2020 revealed an alarming 25% increase in the number of youth in the subclinical or clinical range for internalizing symptoms, and a 39% increase in the number of youth in the subclinical or clinical range for externalizing symptoms, since 2018 [35]. Youth of color were bore the brunt of these mental health consequences: not only did their communities report the highest rates of infection, severe disease, hospitalization, and death from the virus, but they also experienced the greatest economic devastation and were the least likely to recover from it [36–38]. For example, compared to White individuals, racial and ethnic minorities represent a larger share of service and retail workers, who were among the last to regain employment. Indeed, the unemployment rate peaked at 18.5% for Black and 16.7% for Latinx people (versus 14.1% for White people). Relative to Whites, Black and Latinx individuals also reported facing more challenges paying rent and mortgage [39], and faced evictions at higher rates [40, 41]. These disproportionate effects of employment and housing instability likely extended to youth of color [36]. Black and Latinx youth were also more likely to attend schools where at least 75% of students qualify as low-income and food insecure per federal standards, and are therefore eligible for free or reduced-price school meals (Boschma & Brownstein, 2016). COVID-related school closures, therefore, disproportionately constrained Black and Latinx children’s access to the childcare and nutrition typically received in school settings [36, 40, 42]. A cohort study also found that Asian, Black and multiracial adolescents reported greater COVID-related distress and discrimination compared to their non-Hispanic White counterparts [43]. Combinations of these factors are likely implicated in elevated stress levels among youth of color during the pandemic. Importantly, such disparities are not confined to this specific time period, as the economic and structural inequities that exacerbated these issues during the pandemic remain entrenched.

Several studies document mental health concerns among youth during the COVID-19 pandemic [33, 35, 40, 44–50], with some focusing on youth of color [43, 50–64]. However, possible mechanisms undergirding the growing mental health needs of youth of color are understudied. Pre-pandemic research indicates that poverty can elicit depression among youth of color through feelings of hopelessness, helplessness, and limited future expectations regarding economic potential/mobility [30]. The Unified Macrotheory of Depression among Urban Black Youth also offers an explanatory framework: it centers *oppression* as a determinant of *adolescent depression*, mediated by factors including *poverty*, *chronic stress*, and *hopelessness* (Hammack et al., 2003). Additionally, qualitative research has captured the salient role of isolation and loneliness in inner-city Black youth’s experiences with depression [65]. Beyond this work, however, the literature examining the links between mental health, loneliness, and financial insecurity primarily involves older adults and communities outside of the United States [5, 66–71]. For instance, social causation theory has been proposed to explain how exposure to social isolation, among other conditions, may serve as a possible mechanism underlying the effect of poverty on depression. Less is known about whether and how this theory may apply to younger populations [5].

Given these gaps, it is important to examine the psychological pathways through which mental health concerns have developed or intensified among youth of color during COVID-19, especially in light of novel or heightened stressors introduced into their social environments [35, 46, 72–74]. Informed by the prepandemic literature and the Unified Macrotheory of Depression among Urban Black Youth (Hammack et al., 2003), we tested the relationships among COVID-related financial insecurity, stress, loneliness and depressive symptoms with urban youth of color (majority Black). Our investigation was guided by two research questions: [1] Are COVID-related financial insecurity, neighborhood income and free/reduced-price lunch eligibility associated with depressive symptoms among youth of color [2]? Do perceived stress and loneliness mediate the associations between these indicators of economic insecurity and depressive symptoms? We hypothesized that greater financial insecurity due to COVID-19, lower neighborhood income, and free/reduced-price lunch eligibility would each be associated with higher levels of depressive symptoms. Furthermore, we expected these associations to be partially explained by increased levels of stress and loneliness.

## Method

### Participants

We recruited a convenience sample of 105 adolescents and young adults of color from community organizations in Kansas City, Missouri, as part of a broader community-based intervention focused on marginalized youth’s well-being. The present analysis is a secondary examination of survey data collected within that intervention. According to the intervention’s recruitment protocol, participants were eligible if they were aged 13–21, identified as a racial or ethnic minority, and could read and complete a survey in English. We did not independently re-screen participants for our secondary data analysis. The broad age range was selected to be inclusive of the different developmental stages at which youth may encounter economic insecurity. Younger adolescents often perceive financial hardship indirectly, through caregivers’ stress or household instability, which can significantly shape their mental health [75, 76]. Adolescents’ own perceptions of material deprivation may also differ from caregiver accounts and are independently linked to behavioral outcomes [76]. In contrast, young adults are more likely to experience financial strain directly as they transition into greater independence, including responsibilities for managing expenses or housing, which are also strongly tied to mental health [4, 77]. While we did not stratify by age, we aimed to include this full spectrum of youth to reflect how economic insecurity may affect mental health across developmental stages. The mean age of the participants was 16.5 (SD = 1.6). The sample was majority Black (84%) and female (66%). Participant descriptive characteristics can be found in Table 1.

**Table 1:** Participant characteristics

	Total (*N* = 105)
Demographics	
Gender, n (%)	
Female	69 (65.7%)
Male	36 (34.3%)
Age, mean (SD)	16.47 (1.60)
Race, n (%)	
African American/Black	87 (83.7%)
Hispanic American	7 (6.7%)
Asian American	3 (2.9%)
Native Hawaiian/Other Pacific Islander	3 (2.9%)
White	3 (2.9%)
Other	1 (1.0%)
Indicators of poverty and restricted economic opportunity	
Zip code, n (%)	
Low Income (Household income < $43,000)	49 (50.0%)
High Income	49 (50.0%)
Free and reduced lunch, n (%)	
No	50 (50.0%)
Yes	50 (50.0%)
Inability to pay rent/gas/food in the last 2 weeks as a result of COVID-19, n (%)	
No	83 (79.1%)
Yes	22 (21.0%)
Mediating Variables	
Perceived stress, mean (SD)	27.60 (5.88)
Loneliness, mean (SD)	20.54 (15.01)
Outcome	
Depression, mean (SD)	8.12 (6.06)

### Procedure

In May-June, 2020 participants were recruited to complete an anonymous online survey about their experience coping with the COVID-19 pandemic. Our community partners in Kansas City, MO provided the research team with email addresses of affiliated youth in the community. Potential participants were contacted via email, screened for eligibility, and consented to participate via REDCap. Due to Institutional Review Board’s determination of minimal risk, parents/guardians were not consented or informed whether their child participated in the survey. Participants received a $25 gift card for completing the survey. All study procedures were approved by (blinded for peer review) Institutional Review Board.

### Measures

#### Demographics

Demographic variables included gender, age, and race.

#### Indicators of economic insecurity

We examined three indicators of economic insecurity: financial insecurity due to COVID-19, neighborhood income, and free/reduced-price lunch eligibility. Each indicator was analyzed as an independent variable and included as a separate predictor in the path analysis model to capture distinct dimensions of structural versus acute material hardship.

Neighborhood income served as a proxy for community-level socioeconomic disadvantage. It was determined by linking participants’ zip codes to median household income data from the American Community Survey. Zip codes were used instead of census tracts to reduce participant burden and ensure feasibility during remote data collection. Neighborhood income was dichotomized at a threshold of $43,000 based on the median neighborhood income in our sample. While zip-code based measures are broader than census tracts, they provide insight into the structural economic context in which youth reside. We examined the distribution of participants across zip codes and found that most zip codes were represented by only one participant. As a result, there was no meaningful clustering by geographic area, and multilevel modeling was not necessary for the current analysis.

Free or reduced lunch eligibility was measured by a single “yes” or “no” question, reflecting long-term, individual-level economic disadvantage, tied to federal poverty thresholds. This measure is commonly used in adolescent research as an indicator of restricted economic opportunity [78].

Financial insecurity due to COVID-19 was measured using three items adapted from the Centers for Disease Control and Prevention (CDC) COVID-19 Community Survey Question Bank [79]: “In the past two weeks, has your family experienced the following as a result of COVID-19?” Answer options included, “Not enough money to pay rent,” “Not enough money to pay for gas,” and “Not enough money to pay for food.” If participants answered “yes” to any of these three items, they were coded as having reported financial insecurity due to COVID-19. Designed to capture acute household-level financial disruptions during the pandemic, this variable was treated independently from the other two economic indicators. Although this variable reflects household-level disruptions, prior research supports the validity of youths’ self-reported awareness of economic hardship, even among younger adolescents [75, 76]. For older youth, the items may also reflect direct financial burdens, such as challenges with food or housing [4].

#### Loneliness

Loneliness was measured by the Revised UCLA Loneliness Scale, a 20-item self-report measure [80]. Items were assessed on a 4-point scale with response options ranging from 0 (“I never feel this way”) to 3 (“I often feel this way”). Total scores ranged from 0 to 60, where higher scores indicate higher levels of loneliness. Cronbach’s alpha in our sample = 0.96.

#### Stress

Stress was measured using the Perceived Stress Scale (PSS) which assesses the extent to which one experiences stressful thoughts and feelings over the previous seven days [81]. The scale includes 14-items measured on a 5-point scale ranging from 0 (“Never”) to 4 (“Very often”). Total scores ranged from 0 to 56 where higher scores indicate higher levels of perceived stress. Cronbach’s alpha in our sample was 0.71.

#### Depression

Depression was measured by the Patient Health Questionnaire (PHQ-9) [82]. Items were assessed on a 5-point Likert scale with response options ranging from 0 (“Not at all”) to 4 (“Very often”). Total scores ranged from 0 to 36 where higher scores indicate greater frequency of depressive symptoms over the previous two-week period. Cronbach’s alpha in our sample was 0.87.

### Analysis

SAS, version 9.4 was used to run descriptive statistics, including frequencies, means, standard deviations, and correlations. Mplus, version 8.3 was used to path analysis informed by prepandemic literature and the Unified Macrotheory of Depression among Urban Black Youth [83]. All three indicators of economic insecurity—COVID-related financial insecurity, neighborhood income, and free/reduced-price lunch eligibility—were entered simultaneously into the model as independent variables. Each was tested for its association with the mediators (perceived stress and loneliness) and the outcome (depressive symptoms).

Data was examined for degree of missingness and determined: depression (0.95%), stress (0.95%), loneliness (2.86%), free or reduced lunch eligibility (4.76%), and mean neighborhood income (6.67%). Missing data was accounted for using full information maximum likelihood. Bootstrapping analyses with bias-corrected 95% confidence intervals were conducted with 1000 bootstrapped samples to estimate the following indirect associations: (a) each economic insecurity indicator on depression via perceived stress, and (b) each economic insecurity indicator on depression via loneliness. Residual covariances between loneliness and perceived stress, and between the three indicators of economic insecurity, were constrained to zero in accordance with our conceptual framework and to maintain parsimony. As a result, model fit statistics were not reported because the model is just-identified (i.e., number of free parameters is equal to number of known values). Unstandardized and standardized parameter coefficients for total, direct, and indirect associations are reported.

## Results

Sample characteristics are summarized in Table 1. Financial insecurity due to COVID-19 was significantly associated with mean neighborhood income (*r*= −0.20, *p* = 0.046), stress (*r* = 0.22, *p* = 0.02), loneliness (*r* = 0.22, *p* = 0.03), and depression (*r* = 0.26, *p* = 0.01). Additional bivariate analyses identified that stress (*r* = 0.54, *p* < 0.001) and loneliness (*r* = 0.57, *p* < 0.001) were significantly associated with depression. Free or reduced lunch eligibility and mean neighborhood income were not significantly associated with any additional variables in the model.

**Fig. 1:**
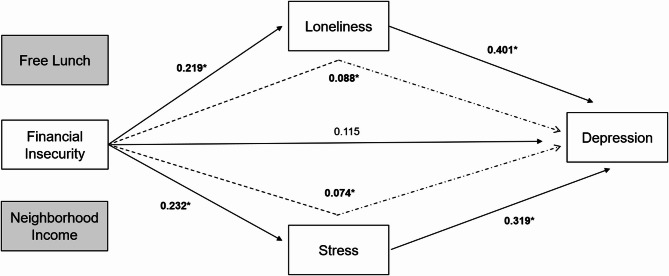
Direct and Indirect Standardized Path Coefficients. Solid arrows indicate direct associations. Dashed arrows indicate indirect associations. Path coefficients from Free Lunch and Neighborhood Income are non-significant and not presented in Fig. 1. *p < 0.05

Based on the Unified Macrotheory of Depression among Urban Black Youth [83], we tested the model depicted in Fig. 1. The pathway from financial insecurity due to COVID-19 and stress was significant (standardized pathway coefficient = 0.232, SE = 0.099, 95% CI [0.025,0.43]), and the pathway from financial insecurity due to COVID-19 and loneliness was significant (standardized pathway coefficient = 0.219, SE = 0.095, 95% CI [0.043,0.407]). The pathways from the two other indicators of economic insecurity – mean neighborhood income and free or reduced lunch eligibility – and loneliness and stress were all non-significant.

The pathway from stress to depression was significant (standardized pathway coefficient = 0.319, SE = 0.095, 95% CI [0.119,0.489]), as well as the pathway from loneliness to depression (standardized pathway coefficient = 0.401, SE = 0.073, 95% CI [0.025,0.545]). There was not a significant direct association from financial insecurity due to COVID-19 and depression (*p* = 0.231), but significant indirect associations were observed from financial insecurity due to COVID-19 through loneliness to depression (standardized pathway coefficient = 0.088, SE = 0.045, 95% CI [0.018, 0.191]) and from financial insecurity due to COVID-19 through stress to depression (standardized pathway coefficient = 0.074, SE = 0.04, 95% CI [0.017, 0.180]). The overall model explained 43.6% of the variance in depression, 5.2% of the variance in stress, and 5.2% of the variance in loneliness.

## Discussion

We applied the Unified Macrotheory of Depression Among Urban Black Youth to investigate the psychological mechanisms through which depression may be associated with financial insecurity and stress during crises such as the COVID-19 pandemic. We found an indirect association of financial insecurity during COVID-19 on depression through pathways of stress and loneliness among youth of color. Extant literature examining causal processes suggests that the link between income inequality and general health are likely to be primarily mediated through psychological stress [84]. Our evidence from the COVID-19 pandemic demonstrates that a similar understanding may also be applied to the development of depression. This link between stress and depression is consistent with prior work demonstrating that exposure to multiple stressors is associated with increased depressive symptoms among adolescents (85, 86). Such an association likely extend beyond the context of the pandemic and reflect broader patterns of structural inequity disproportionately affecting youth of color (87).

In addition to stress, our analysis suggests that loneliness is another crucial factor in the relationship between financial insecurity and depression. While loneliness is recognized as an important contributor to mental health problems [66–71], there are limited investigations with youth of color in the United States. This warrants future research that examines how loneliness impacts development, and characterizes loneliness among youth of color. Furthermore, interventions to address loneliness largely focus on social and emotional learning [84], and less on the economic conditions that influence youth development. Given the current paucity of literature, there is a greater need for researchers to further explore loneliness as a mediator in the relationship between financial insecurity and depression, particularly among youth of color who disproportionately experience poverty.

In interpreting the results, it is important to consider the conceptual distinctions among the three economic security indicators used in our analysis. Although all three indicators are theoretically important, only financial insecurity due to COVID-19 was significantly associated with stress, loneliness and depressive symptoms in our analysis. One potential explanation is that this measure captures recent and acute disruptions to participants’ daily lives, such as the inability to afford food, rent or transportation, during an already stressful and uncertain period. Because our data rely on self-reported perceptions, the personal salience of these acute hardships may have also played a key role in how youth interpreted and reported their experiences. For example, financial insecurity due to COVID-19 captures recent, household-level disruptions such as difficulty paying for rent, food, or transportation—conditions likely to be more psychologically salient in a self-report survey. This personal salience may explain its stronger associations with stress, loneliness, and depression in our model. On the other hand, neighborhood income served as a proxy for structural and environmental disadvantage and was derived from median household income within participants’ zip codes using publicly available census data. While this measure captures community-level context, it may not reflect youth’s direct or perceived experience of hardship, particularly in a crisis context such as the pandemic. Free/reduced-price lunch eligibility similarly represents a structural indicator of long-term poverty exposure, but may not have varied meaningfully within our sample, as 50% of participants reported receiving free lunch and attended schools with high concentrations of low-income students. These findings underscore the need for future research to closely examine how both chronic and acute forms of economic insecurity intersect and are perceived by youth, particularly during crisis periods.

This study includes some limitations. First, its cross-sectional design limits causal inference. As such, we rely on social theory to inform the order in which variables are presented. Second, “Hispanic” appeared in both the race and ethnicity variables in the original survey used in the intervention. We retained the race variable as the primary classification to ensure consistency, reduce misinterpretation, and maintain mutually exclusive groupings for analysis. Third, the dataset included three participants who self-identified as White, despite the intervention’s stated eligibility criteria. We retained these participants to preserve the integrity of the original dataset and avoid post-hoc exclusion, but acknowledge this is a minor deviation from the intended inclusion criteria. Fourth, our measure of neighborhood income, based on zip code-level medians and dichotomized at the sample median, does not reflect absolute poverty or federal thresholds, and may misclassify some areas as “not disadvantaged” despite persistent structural inequities. Finally, the small sample involving predominantly inner-city Black youth limits the generalizability of our findings to other groups. Future investigations of this topic will benefit from a longitudinal design and a larger sample with greater geographic and racial/ethnic diversity.

Despite these limitations, the current study highlights the importance of curtailing depression among youth of color through economic security interventions, both during crises like the COVID-19 pandemic and as part of long-term public health strategies. These interventions should comprehensively address the stressors associated with financial insecurity and loneliness as an innovative approach to mental health promotion among youth of color. This approach would align well with current calls for policy research that examines the role of economic security interventions on health and well-being of populations of color who are disproportionately impacted by concentrated poverty and declines in physical and psychosocial health. These approaches may hold promise in addressing the triple-jeopardy of financial insecurity, stress, and loneliness when addressing the mental health needs of youth of color, not only to address the aftermath of COVID-19, but also lay the foundation for sustainable support systems for youth of color in the post-pandemic era.

## Data Availability

De-identified data from this study are not available in a public archive. Requests to access de-identified data can be sent to the corresponding author, but will require approval from IRB and community partners.
